# Automatic stochastic 3D clay fraction model from tTEM survey and borehole data

**DOI:** 10.1038/s41598-022-21555-z

**Published:** 2022-10-12

**Authors:** Alexis Neven, Anders Vest Christiansen, Philippe Renard

**Affiliations:** 1 Centre of Hydrogeology and Geothermics, University of Neuchâtel, Neuchâtel, Switzerland; 2 Department of Earth Sciences, Aarhus University, Aarhus C, Denmark; 3 Department of Geosciences, University of Oslo, Oslo, Norway

**Keywords:** Geophysics, Hydrogeology

## Abstract

In most urbanized and agricultural areas of central Europe, the shallow underground is constituted of Quaternary deposits which are often the most extensively used layers (water pumping, shallow geothermic, material excavation). All these deposits are often complexly intertwined, leading to high spatial variability and high complexity. Geophysical data can be a fast and reliable source of information about the underground. Still, the integration of these data can be time-consuming, it lacks realistic interpolation in a full 3D space, and the final uncertainty is often not represented. In this study, we propose a new methodology to combine boreholes and geophysical data with uncertainty in an automatic framework. A spatially varying translator function that predicts the clay fraction from resistivity is inverted using boreholes description as control points. It is combined with a 3D stochastic interpolation framework based on a Multiple Points Statistics algorithm and Gaussian Random Function. This novel workflow allows incorporating robustly the data and their uncertainty and requires less user intervention than the already existing workflows. The methodology is illustrated for ground-based towed transient electromagnetic data (tTEM) and borehole data from the upper Aare valley, Switzerland. In this location, a 3D realistic high spatial resolution model of clay fraction was obtained over the whole valley. The very dense data set allowed to demonstrate the quality of the predicted values and their corresponding uncertainties using cross-validation.

## Introduction

Shallow alluvial Quaternary aquifers are frequently used for water supply or shallow geothermal energy exploitation. In this context, a wide range of associated questions often needs to be addressed, such as evaluating groundwater resources, studying contaminant migration, evaluating interactions with surface water, or avoiding an overlap of influence zones between neighboring geothermal wells. All of these questions can only be properly answered after modeling the structure and internal heterogeneities of those aquifers.

These models are often constructed in several steps^1,2^. For small-scale models, the use of boreholes as the only source of data is common. However, such an approach is often blind to most of the spatial heterogeneity and thereby may lead to inadequate models and wrong conclusions. Boreholes are only one source of information to infer the local and vertical distribution of the facies. They are often of limited help to estimate complex 3D structures. When the area of interest is wide, increasing the number of boreholes to reduce the uncertainty to an acceptable level is often difficult, time-consuming, and expensive. A solution is to combine less expensive geophysical data and borehole data. Geophysical data are often interpreted and combined manually into a structural model, which is then filled with lithological or facies simulations and finally with physical properties. Such a workflow has proven to be efficient in generating geological models from aerial electromagnetic or seismic data for example^3,4^. But these modeling steps are complex and often need different software. Furthermore, even if some stochastic methods are often used^4,5^, the uncertainties are not always propagated through the complete workflow. Often some steps are considered deterministic and the resulting geological model is the one that fits most of the comprehensive knowledge available^6–9^. Finally, when working with a large dataset, the manual construction of the structural model using both boreholes and geophysical data can be time-consuming.

Therefore, there is a need for an automatic approach that would be able to integrate multiple data types and produce structural or parametric models. For example, the fast generation of 3D clay models with an automatic algorithm could be of great benefit to local authorities who often do not have the capacity to run manual integration of the data.

However, in most cases, the main data available along boreholes are lithological descriptions and not resistivity or density values. But these physical parameters are the ones infusing the geophysical data. Linking directly resistivities and lithologies is difficult due to the wide variety of factors affecting resistivity^10^, and the often incomplete description of the lithological facies. The most fundamental relationship is Archie’s Law^11^, linking resistivity to saturation, water conductivity, tortuosity, and porosity. This empirical relationship is based on the assumption that the matrix is non-conductive, an assumption not valid as soon as we have the presence of clay minerals conducting current at their surface. This pore-surface conductivity will depend on the surface area, grain size, clay type, and clay content. Estimation of all these parameters that can have a high spatial variability is tricky. A recent review^12^ points out scale issues as well. Most of the laboratory empiric laws are measured at a core scale, where the sample is in the range of a dozen of cubic centimeters. And, the upscaling of such laws to the field scale is not straightforward. Finally, most of the lithological descriptions associated with boreholes are qualitative and not quantitative^12^. Also, it is way too simplistic to apply a function that would link the lithological description to a single resistivity. Each lithological description can be associated with a wide range of resistivity with some overlap between different lithologies^13^.

An answer to that issue is to define the probability of having a given lithological facies conditioned on the resistivity value. These probability distributions can be estimated from a sampling of boreholes and coinciding resistivity models^13^. However, such an approach does not take into account the spatial distribution of the boreholes and ignores the possible spatial dependence between the type of sediment and its resistivity. The Probability Distribution Function (PDF) is calculated from boreholes and resistivity models all over the domain. If a given lithological facies is always more resistive in a subarea of the domain, the PDF calculated over the whole domain will not reflect this.

To solve that issue, instead of trying to estimate a single PDF, Foged et al.^14^ proposed a method based on the inversion of a spatially varying translator function between resistivity and CF. The function gives the best fit between the observed CF in boreholes and the CF computed from the resistivity models. This method has the advantage of not relying on any prior parameter estimation and is only inferred from observed data. It also has the advantage of transforming the lithological description to a continuous variable making the upscaling possible, while taking the co-location of the function into account. However, even if this method can estimate the CF at the position where geophysical resistivity models exist, it still needs to be interpolated to obtain a full 3D continuous model.

To this extent, after predicting the CF, Vilhelmsen et al.^15^ used a geostatistical approach. Various geostatistical methods exist^16–18^ and are capable of interpolating data and producing realistic models and simulations. Their use is widespread in disciplines such as risk assessment, resources management, mining, petroleum engineering, or geological modeling. Multiple Point Statistics^19,20^ (MPS) is one of these geostatistical techniques. It is a non-parametric method that relies on the use of a Training Image (TI) to infer the spatial variability of one or multiple variables. MPS has proven to be capable of generating realistic complex spatial variability in a broad of situations^21–25^. Vilhelmsen et al.^15^ clustered the CF in units and used it as TI in an MPS procedure. The Hard Data were defined as being the zones where the cluster is the most certain, mainly at really low and high resistivity. The other areas are only constrained by soft data. This allows one to reflect one type of uncertainty on the CF data and to show variations in uncertain model zones. But this method requires choosing the discrete threshold value at which the belonging to a cluster becomes certain. In addition, it does not allow the propagation of uncertainty from the data. The uncertainty on resistivity or on CF is lost after the clustering. Therefore, a value that would be close to the cluster limits but associated with a large uncertainty will be considered as certain as a value that would enter a cluster beyond any doubt. Moreover, due to the shape of the translator function, small changes in resistivity value in the transition zone between clay and non-clay can have huge impacts on the estimated CF. Finally, since the uncertainty of the data is used as normalization in the objective function of the CF inversion, we argue that it should also be considered when applying the function and interpolating the results. This shows the limitation of using a deterministic TI when the uncertainty should be taken into account.

In this paper, we propose an extended workflow to automatically generate a 3D clay fraction model, with a robust uncertainty propagation from the data to the final model. The paper is structured as follows. We first present the three main steps of the methodology: the CF deterministic inversion, the stochastic interpolation framework, and finally the cross-validation implementation. Then, we present the application of the methodology for a ground-based towed Transient Electromagnetic (tTEM) geophysical dataset^26^ acquired in the upper Aare valley, Switzerland.

## The automated workflow

**Figure 1:**
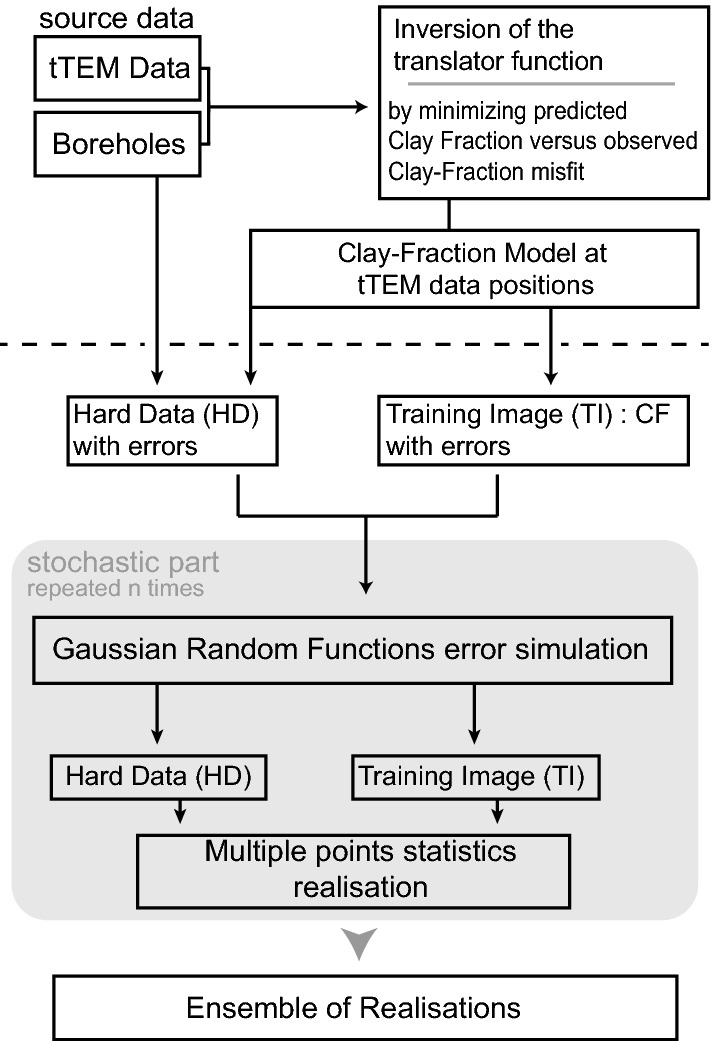
Overview of the mains steps of the proposed methodology. The shaded area highlights the stochastic part of the workflow.

The proposed methodology allows generating automatically a 3D CF model of a complete Quaternary valley. A key aspect of this approach is to rely on the data itself to infer most of the parameters automatically. The following sub-sections of this paper will describe in detail all the steps. But, let us first provide a rapid overview of the complete workflow as outlined in Fig. 1.

The inputs are, on the one hand, a dense data set of resistivity logs obtained by inverting the geophysical measurements (here tTEM data) and, on the other hand, a much sparser data set of geological logs which can be used to estimate the reference CF along the boreholes. To produce a 3D CF model over the whole domain, we first invert a CF Translator Function (Step 1 in Fig. 1) using the method of Foged et al.^14^ and both data sets. The translator function is then used for estimating the CF from the resistivity for all the available geophysical soundings (including locations where no borehole data is available). The main novelty of the proposed workflow is the methodology employed to interpolate the resulting CF values in 3D (Step 2 in Fig. 1). As compared to previous works, there are two major improvements. One is to avoid having to classify the data; the CF values are used directly. The second, and maybe most important, is that we consider the various sources of uncertainties and propagate them in the complete workflow. For this, the interpolation is done with a Multiple Point Statistics algorithm, using the CF data themselves in 3D as a Training Image (TI). The dense geophysical coverage permits using directly the data without having to add external information. To account for uncertainties in both the boreholes and the tTEM data, we use Gaussian Random Functions to simulate error maps, that are included in the conditioning data and TI images prior to the MPS simulations. Finally (Step 3 in Fig. 1), a cross-validation is performed to check the quality of the results. A part of the conditioning data are not used during the interpolation and compared with the results. We then assess how the method performs both in terms of predicted value and predicted uncertainty.

### Clay fraction estimation

This part of our methodology (Step 1 in Fig. 1) follows closely the work of Christiansen et al.^27^ extended by Foged et al.^14^. We apply their idea of inverting a function that fills the gap between resistivity and CF. Since the method has been described in detail in these previous publications, only a brief summary is presented here. This approach was first developed to assess nitrate contamination risk^27^ and called Accumulated Clay Thickness (ACT). Later, an extension was proposed to estimate the CF with the same methodology^14^. The main assumption is that the major changes in resistivity in saturated and unconsolidated deposits are caused by variations in the amount of clay. With this in mind, a translator function linking the resistivity and the CF can be constructed.

Although, a few challenges need to be discussed. First, the resistivity models derived from the geophysical data are always a smoothed version of the reality, because of the footprint of the instrument, the resolution capability, and the inversion procedures. Second, the composition of the clay sediment itself can change and cause different resistivity values in different places with similar lithological descriptions. This implies that a translator function based using only resistivity as an input cannot represent properly the link between resistivity and hydraulic conductivity.

**Figure 2:**
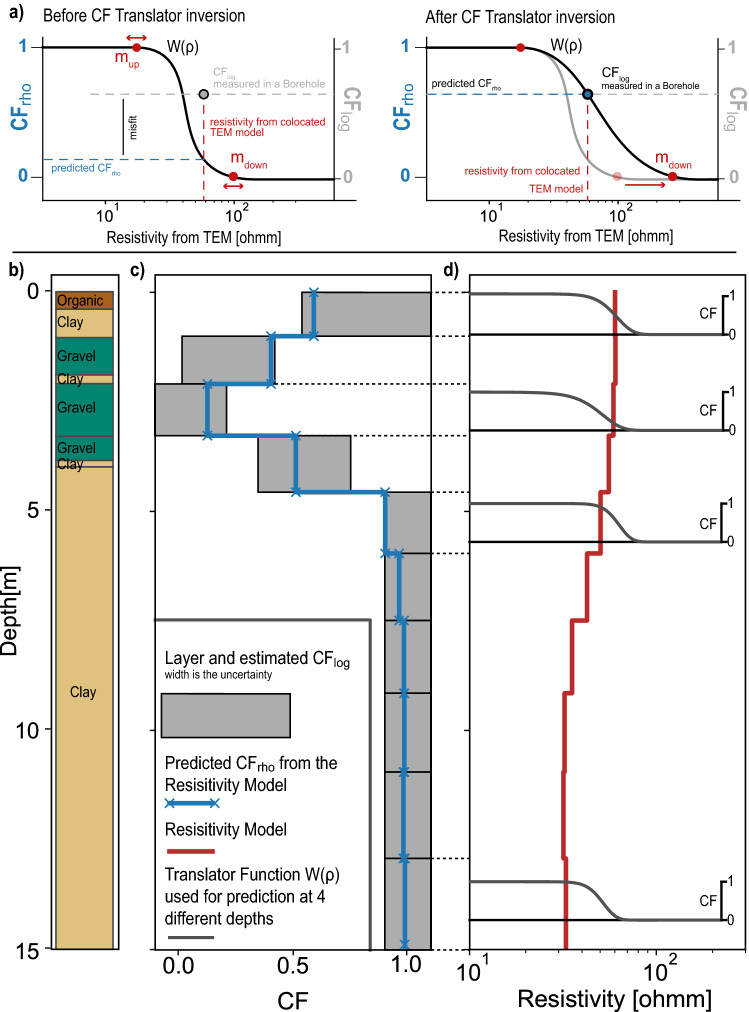
(**a**) Illustration of the automatic fit of the translator function on one data point. (**b**) Example of a USCS principal component lithological log. (**c**) Associated estimated CF in a regular layered grid ($CF_{log}$), derived from the lithological description of the borehole. The line is the predicted CF using the resistivity models and the inverted translator functions. ($CF_{rho}$) (**d**) Resistivity model from the smooth inversion of the tTEM data at the borehole position. For four layers, the inverted translator function used is displayed.

Foged et al.^14^ therefore proposed a procedure based on the inversion of two parameters of a translator function that predicts a CF from a resistivity value and allows these parameters to vary in space. In other words, two identical resistivity values that are far from each other will not necessarily correspond to the same CF value. The basic input data, in this procedure, are boreholes geological logs as illustrated in Fig. 2b and geophysical resistivity profiles at the same locations such as the one shown as a red curve in Fig. 2d. The two data sets have been acquired using independent methods and are co-located.

Based on the geological description, the clay fraction $CF_{log}$ along the borehole is estimated by dividing the geological log into regular depth intervals and by computing the proportion of lithological clay for each interval. Because the geological description is often qualitative, the resulting CF is uncertain and the result is a mean value and range of CFs for each interval. This result is illustrated as gray boxes in Fig. 2c.

The next step is to define a parametrized translator function:

1 $$\begin{aligned} W(\rho ) = 0.5 \cdot \text {erfc} \left[ \text {erfc}^{-1}(0.05) \cdot \frac{ 2\rho - m_{up} - m_{low}}{m_{up} - m_{low}} \right] \end{aligned}$$

where *erfc* is the complementary error function, $\rho$ is the resistivity, and $m_{up}$ and $m_{low}$ are the resistivity values at which the function returns a weight of 0.975 and 0.025 respectively. The parameters $m_{up}$ and $m_{low}$ are identified during the inversion and they can be thought of as the resistivity limits for only clay/only sand. Figure 2a illustrates the inversion of $m_{up}$ and $m_{low}$ for one data point. Before the inversion, the predicted $CF_{rho}$ is in disagreement with the observed amount of clay $CF_{log}$ described in the collocated borehole, resulting in a misfit. The inversion will adjust $m_{up}$ and $m_{low}$ to reduce the misfit.

The previous example shows the principle for one single data point, but the problem is correlated in 3D and aims at minimizing a global misfit. Two neighboring translator functions cannot have drastically different parameters. Figure 2d shows for example how the translator function will vary with depth for one single borehole. The function is outlined for four different depths in Fig. 2d, but is defined for all layers.

To obtain this type of result, the inversion procedure follows the following steps. First, the global data residual is defined as the average squared error between the predicted CF ($CF_{rho}$) and the measured CF in the boreholes ($CF_{log}$):

2 $$\begin{aligned} R_{dat} = \frac{1}{N} \cdot \sum _{i = 1}^N \frac{(CF^{rho}_i - CF^{log}_i)^2}{\sigma _i^2} \end{aligned}$$

The data residual is normalized by $\sigma ^2$, the combined variance of $CF_{rho}$ and $CF_{log}$, and *N* the number of conditioning data. Regularization parameters are then added to ensure that neighboring points do not show sharp and unrealistic variations. The regularization will limit the spatial variation of $m_{up}$ and $m_{low}$. The regularization is expressed as:

3 $$\begin{aligned} R_{con} = \sqrt{\frac{1}{N_{con}} \cdot \sum ^{N_{con}}_{i=1} \frac{A_i^2}{ln(e_i)^2}} \end{aligned}$$

where $N_{con}$ is the number of pairs of parameters, *A* is the difference in log space between the two pairs of parameters investigated, and $e_i$ is a distance-dependent factor. The further away two points are, the larger can their difference be. Finally, the complete objective function is:

4 $$\begin{aligned} Q = \sqrt{ \frac{ N \cdot R_{dat} + N_{con} \cdot R_{con} }{N + N_{con}}} \end{aligned}$$

The optimal parameters ($m_{up}$ and $m_{low}$) are obtained by minimizing *Q* using an iterative Gauss-Newton scheme with a Marquardt modification^27^. The fit between the predicted $CF_{rho}$ and $CF_{log}$ is not perfect, since the translator function parameters are affected not only by the co-located data (borehole-resistivity models) but also by all the neighboring ones (via the regularization). The inversion looks for a global minimum.

In the example shown in 2, we can denote how the translator function adapts to identify structures that are not well represented in the resistivity model, such as the shallow clay layer.

At the end of the clay fraction estimation step, each resistivity log is associated with an estimated $CF_{rho}$ log, obtained by applying the translator fonction with the optimal parameters $m_{up}$ and $m_{low}$.

The final step is to propagate the uncertainty from the resistivity values to the CF. Indeed the resistivities were obtained by a geophysical inversion which is capable of estimating the uncertainty on the resistivities. The translator functions can then be applied not only to the resistivity data but also to their uncertainties, resulting in a $CF_{rho}$ at the tTEM acquisition points with uncertainty. For the next simulation step, the $CF_{log}$ will be used as conditioning data for the cells in which we have borehole information. $CF_{rho}$ will be used elsewhere.

### 3D stochastic interpolation

To generate a full 3D model of CF and resistivity, the values obtained in the previous step need to be interpolated to cover the space where no geophysical or borehole data have been acquired. This part of the workflow corresponds to Step 2 in Fig. 1 and is the main novel part of the proposed workflow. We use the Direct Sampling MPS method^28^ in this process. The main advantage of using an MPS approach is that it can learn automatically the spatial patterns of the regional structures from the very dense data set constructed in the previous step and use it to represent the spatial variability and uncertainty in the interpolated areas. However, the standard application of MPS techniques assume that the TI and the Hard Data (HD) are deterministic. Here, we propose a method to go a step further and account for uncertainty in these input data. We therefore include non-deterministic Hard Data (HD) and Training Images (TI) in the MPS algorithm. The overall method, as shown in Step 2 in Fig. 1, will consist in applying the MPS algorithm many times with different TI and different HD to generate an ensemble of interpolated 3D models of CF. From this ensemble of simulations, we will derive probability distributions for the CF and resistivity at any location in the 3D domain.

#### Direct sampling algorithm

The general principle of MPS algorithms is to fill a simulation grid iteratively while reproducing the patterns of the TI. The Direct Sampling algorithm^28^ is a versatile MPS algorithm based on a randomized and conditional re-sampling of the TI. In this paper, we use the *Deesse* implementation^29^ of the Direct Sampling. To generate one simulation, the 3D grid is first filled with the Hard Data available. The algorithm then randomly visits all the remaining locations in the grid. For simulating a value at a given location, the *n* closest HD and already simulated values are extracted to define a data pattern. The data pattern is then used to search for locations having similar patterns in the TI. During that search, the distance between the data pattern and the patterns found in the TI is computed. If the distance is below a threshold value (*t*), then the two patterns are considered similar and the pixel value at the missing location is taken from the TI and copied in the simulation grid. To accelerate the algorithm and avoid copying and pasting directly the TI, only a fraction (*f*) of the TI is scanned. The three parameters *n*, *t*, and *f* are chosen in advance by the user. Meerschman et al.^30^ offer some practical recommendations for the selection of those parameters. One strength of the Direct Sampling algorithm is that it can deal with continuous and multiple variable simultaneously. It means that one can provide a TI containing several variables, and the algorithm can simulate one or several variables conditioned to the other data available. This is described in detail in several papers^28,31^. It can be used to describe the presence of trends in the training image and in the interpolation grid using auxiliary variables^22,24^.

To interpolate the CF and resistivity, we used DeeSse and designed a four variables simulation problem: two auxiliary variables and two main variables. Each of them has a specific number of neighbors *n* and threshold *t*, while the scanned fraction *f* is common to all variables. The two secondary variables are (1) the depth of the cell and (2) the northing of the cell. Their purpose is to describe our prior knowledge of spatial trends. This choice reflects our expectation to find certain patterns more preferably at a given depth or area in our interpolated domain. For example, the deepest structures in the TI will tend to be reproduced deepest in the simulation. However, the threshold values that we selected are high, and *n* is small constraining only midely the algorithm. We also chose to include only northing, since we expect to have most of the variations of the pattern along the valley (oriented roughly N–S). This assumption is based on a visual inspection of the resistivity models, geological knowledge, and boreholes. The main simulation variables are (3) the logarithmic transform of the resistivity and (4) the clay fraction. For these variables, we chose a higher number of neighbors *n* and a smaller threshold *t* as these are the variables of interest. All four of these variables are simulated. We also activate the Gaussian Pyramid option of the DeeSse code^29^. By doing so, the spatial patterns in the data are analyzed and modeled at multiple scales. The algorithm uses Gaussian filters to create a pyramid of co-located coarser scale images that are used jointly for training and simulation^32^. This method improves the quality of the simulation between densely covered areas and sparser ones while being able to be sensitive to different scales of variation.

#### TI and HD generation

To run the MPS algorithm and interpolate the CF, we need to provide a 3D TI and HD. The HD are simply the 3D punctual data derived from Step 1 in the boreholes and geophysical soundings. Because the spatial density of the tTEM data is very high (see the example application below), this data set is often very dense and it can be used directly as a TI. This situation corresponds to the so-called gap-filling problem, in which we need to interpolate only some parts of an already very dense data set. MPS and, in particular, direct sampling have proven to be very efficient for these problems^31,33^. In these cases, the same data are used as HD and TI. The assumption behind this modeling decision is that the coverage of the data is sufficient to represent properly the spatial statistics of the variables that need to be interpolated.

In practice, the 3D HD points are placed in the 3D simulation grid upfront. These points are estimations of CF and resistivity values coming either from the boreholes or from the application of the translator function on the tTEM data. The auxiliary variables (depth and northing) are also computed and stored for every location in the grid. At this point of the methodology, we could simply apply the MPS algorithm and obtain an ensemble of stochastic simulations representing the uncertainty due to the interpolation. This is what is normally done when using MPS. The TI and the HD are deterministic.

But the CF and the resistivity values have an associated uncertainty that has already been estimated in the previous step of the workflow. To account also for this source of uncertainty, a possibility would be to consider only the most certain points as HD, for example, the extremely low or high resistivities, which are almost certainly associated with entirely clay or non-clay points, respectively. But this idea has two main disadvantages. First, a choice has to be made to determine the upper and lower resistivity boundaries. This would go against the idea of implementing the most automated procedure possible. Second, the probability distribution of the values in the TI must be similar to the HD distribution. If they are different, the simulations may tend to over-represent the clay and non-clay points in the simulation. More generally, we would not be using all available HD, and we could assign a resistivity or a CF to a cell completely outside the uncertainty range derived from the field measurements. We, therefore, need a better way to account for the uncertainty on these data.

We overcome these challenges by a combined use of MPS and Gaussian Random Function (GRF) which allows us to perform MPS with a training image (TI) and a hard data set (HD) that are not deterministic. GRF models are well known^18,34^. The spatial variability is modeled using parametric multi-Gaussian distributions. These models are defined with a covariance or variogram model representing the spatial variability. Multiple realizations can be generated, with or without conditioning data. In the proposed methodology, the GRF model is used to represent the CF and resistivity data measurements uncertainty. In practice, for each MPS simulation of CF and resistivity, a different TI and HD are generated.

**Figure 3:**
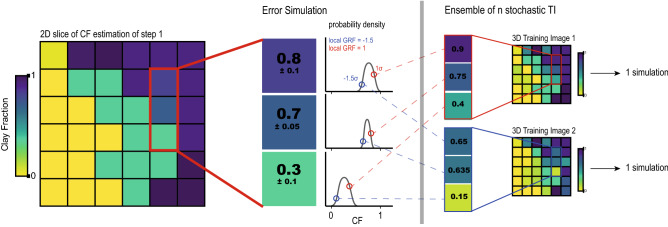
Principle for the generation of an ensemble of Hard Data and Training Images to account for the uncertainty on the HD.

Figure 3 sketches the general principle of the generation of the TIs. The original HD (and TI) are slightly perturbed by adding some noise within the range of estimated uncertainty for these data points. The simulation of the noise is made using unconditional 3D GRF simulations. The GRF simulations have a mean of 0 and a variance of 1. To account for the possible spatial correlation of the noise a 3D anisotropic variogram is automatically fitted to the TI data. We use a Trust Region Reflective algorithm^35^ to optimize the sill and the multi-direction ranges of two contributions (a Gaussian and an exponential). The sill is then normalized to obtain a variance of 1. The values of the CF and resistivity for the 3D TI (and 3D HD) at each iteration will then be defined by adding the HD with a rescaled noise:

5 $$\begin{aligned} \text {TI}_{i}(x,y,z) = \text {data}(x,y,z) + \sigma (x,y,z) \cdot \text {GRF}_{i}(x,y,z) \end{aligned}$$

with $\text {TI}_{i}(x,y,z)$ being the TI value for the simulation *i* at position (*x*, *y*, *z*) in 3D, $\text {data}(x,y,z)$ being the deterministic estimated value of the variable of interest at that location, $\sigma (x,y,z)$ the standard-deviation of the estimated uncertainty and $GRF_{i}(x,y,z)$ being the unconditional simulated GRF value at position (*x*, *y*, *z*) for iteration *i*. The final simulated value (CF for example) varies within the range of its uncertainty. In the example presented in Fig. 3, the initial TI derived from the estimated CF is slightly modified by adding a correlated random noise that depends on the local uncertainties. The same operation is performed for the geophysical and borehole data sets using the same GRF model.

To summarize, one unique 3D TI and its corresponding 3D HD set are generated for each simulation and given as input to the Direct Sampling algorithm which will simulate the missing parts in the 3D grid. This will result in an ensemble of 3D realizations. We can then stack all the simulations and calculate the mean and standard deviation for any location. The cells in which we have conditioning data will have the same mean and standard deviation as the original conditioning data.

#### Cross-validation

To check the performance of the proposed methodology, we implemented a cross-validation step. Cross-validation allows for quantifying the errors associated with a model. The principle is simple: a subset of data is created; it contains a random part of the original dataset. The stochastic interpolation is then applied using only the subset of data as HD, and the resulting simulation is compared with the excluded data. Various error indicators can be used^36^. In our case, a random sampling of the dense CF data does not create sufficiently large gaps to produce representative error estimation. The missing points are too well constrained by the neighboring ones. To create a larger disruption in the data, we assigned each point a group, based on a 3D k-means clustering^37^. The clustering is done using the spatial coordinates (x,y,z) of the model. The purpose of this step is to create multiple spatial groups of similar sizes that will be randomly excluded from the simulations. In our case, we defined 28 groups. The simulated value and the standard deviation of these zones are then compared to the real value. The error and the normalized error for each point in the model are defined as

6 $$\begin{aligned} \varepsilon= & {} \frac{1}{n} \sum _{i=1}^{n} (sim_i - true) \end{aligned}$$

7 $$\begin{aligned} \varepsilon _{norm}= & {} \frac{1}{n} \sum _{i=1}^{n} \frac{\mid (sim_i - true) \mid }{\sigma } \end{aligned}$$

with $\varepsilon$ and $\varepsilon _{norm}$ being respectively the error and the normalized error, *n* the total number of simulations, *true* is the data point not included in the interpolated dataset, $sim_i$ is the simulated value and *sigma* is the standard deviation of $sim_i$ over the *n* simulations that didn’t include these data points. These two indicators are calculated point-wise. The normalized error is an important indicator since it shows how well we estimate the uncertainty, the objective being a ratio close to 1.

**Figure 4:**
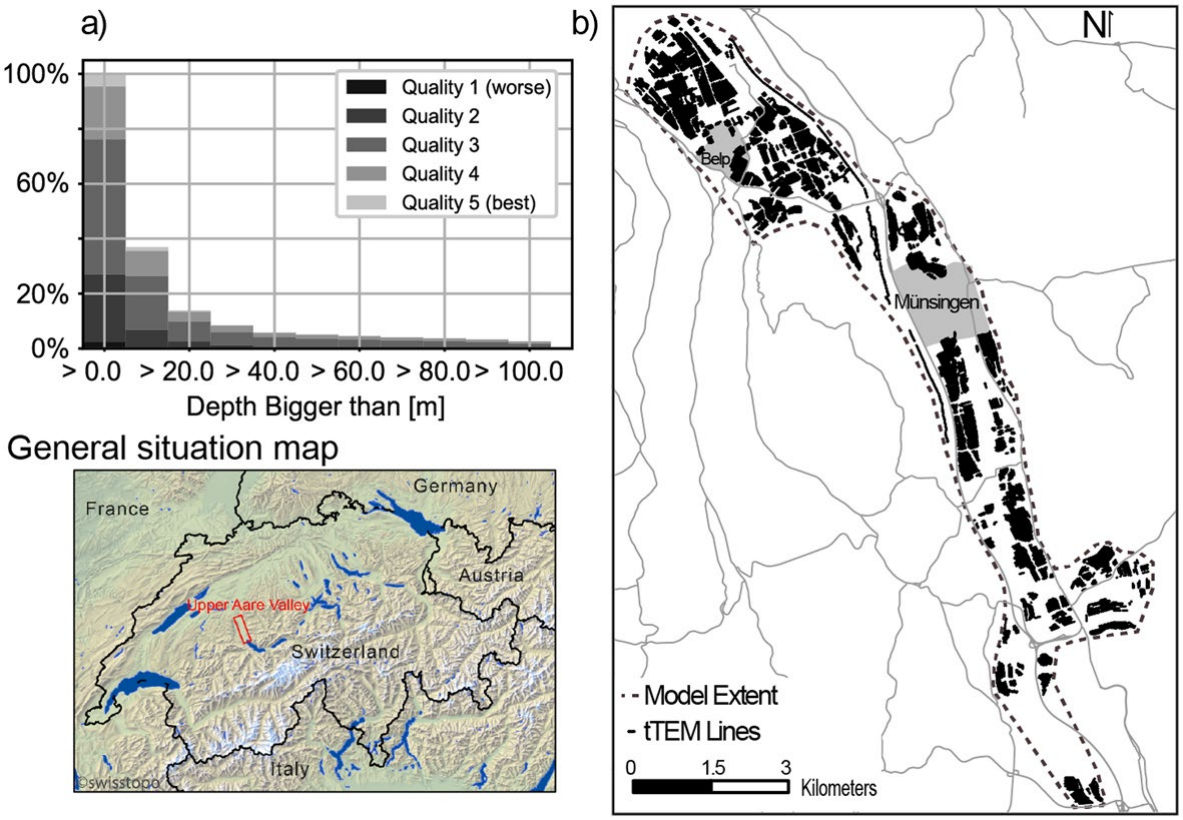
(**a**) Distribution of layers quality and depth after normalization. (**b**) Overview of the coverage of the tTEM data and general situation map (created with QGIS V3.22.9, qgis.org. Basemap is freely available from the Swiss Federal Office of Topography swisstopo).

## Application on the Aare Valley

### Geological settings

The study area is located in central Switzerland and covers a section of approximately 20 km of the Upper Aare Valley between the cities of Thun and Bern. The Upper Aare Valley presents a typical Quaternary geology for alpine valleys: the basement is usually a few hundred meters deep and is covered with a complex interwinding of glacial, fluvio-glacial, and fluvial deposits^38,39^. Multiple cycles of glacial advances and retreats have been identified in the Swiss basin, causing multiple changes in the deposition processes^40^. In the particular case of the Upper Aare Valley, at least four glacial cycles have been identified in boreholes^41^. However, a complete description of the lithostratigraphy based only on borehole descriptions is nearly impossible because similar deposits of different ages may be superimposed and intertwined. A surface aquifer constituted of the most recent alluvial deposit is present all over the valley. The water table is between a few dozen centimeters up to 2 meters below the surface. General knowledge of the aquifer suggests that the thickness of the upper aquifer ranges from 4 to 20m depending on the area. In addition, a second deeper aquifer has been identified in some deep boreholes separated from the shallow one by a clay layer. Both aquifers’ exact extend, connectivity, and thicknesses are not extensively known. However, in the area, 6350 pumping wells (shallow geothermal or drinking water), and 5300 injection wells (re-inject water after geothermal heat pump) are in use. In this context, the realization of a 3D model would be a great benefit for local authorities to evaluate the vulnerability of the upper aquifer. Because of the water table height, saturated conditions can be assumed in almost the entire height of the model.

### Borehole data

In the zone of interest, 1542 boreholes are lithologically described. The upper Aare Valley was also one of the test sites retained for the Geoquat project of the Swiss National Topographic Institute^39^. In this context, they performed digitization and standardization of the borehole data. All boreholes descriptions were converted to standard USCS descriptions^42^. In addition, a QC value was added for each layer assessing the reliability of the geological information: a grade from one to five was assigned to each layer, depending on the precision of the description. one corresponds to a layer with only a basic description when five corresponds to a layer where lab measurements have been performed and a complete multi-phase lithological description is available. An estimation of the Clay Content in the boreholes was made using the USCS guidelines, and the uncertainty was scaled according to the grade. A poorly described clay layer (Quality 1) will have, for example, a CF value of $1 \pm 0.5$. We consider that up to 50% of the clay layer could be non-clay material. On the other hand, a well-described one (Quality 5) will have a CF value of $1 \pm 0.08$. Missing, artificial, or undescribed layers are not taken into account.

Figure 4a shows the distribution of the quality of the layers and their associated depth. Since most of the borehole exploration is conducted for either shallow geothermal exploration or geotechnical purposes, we observe that around 60% of the data are above 10m depth and that the proportion follows roughly a decreasing power-law distribution with depth.

### tTEM data

In January 2020, a ground-based towed transient electromagnetic (tTEM) survey was conducted in the upper Aare valley. The data were inverted using different regularizations (sharp and smooth), and the resulting resistivity models are used in this study. All processing workflow, details about regularization, and data are described in Neven et al.^26^. The dataset covers about 1500 hectares, with a line spacing of 25 meters. The sampling frequency after processing is about 1 resistivity model every 10 meters along the lines. The inversion of the data was done using the *AarhusInv* (https://hgg.au.dk/software/aarhusinv) inversion code^43^. The average residual of the inversion is 0.52 for the sharp regularization, which means that we tend to have an excellent fit between the predicted data from our resistivity models and the field measurements. To estimate the depth of investigation, we used the Jacobian sensitivity matrix of the last iteration^44^. By doing so, we can identify the exact depth at which each resistivity model is only poorly represented in the recorded data. Below this depth, the resistivity models are blinded. Again, for a complete description of the inversion of the data set and the quality check, the readers are referred to Neven et al.^26^.

The translator function inversion was performed on the *tTEM20AAR* sharp inversion dataset^26^. 57’862 resistivity models of 30 layers were taken into account and blinded at the standard depth of investigation. Figure 4b outlines the geophysical data coverage in the valley after processing. Most of the fields outside the cities are mapped. Cities, as well as the areas surrounding roads and train line, are clearly visible since the electromagnetic coupling in such environments forbids the use of an inductive method and are left uncovered. The uncertainty on the resistivity was estimated using the last iteration covariance matrix^45^. Even if this method does not fully replace the uncertainty that can be derived from a stochastic inversion, it has the advantage of being able to reflect the relative uncertainty on the model’s cells while being relatively fast to calculate.

### Clay fraction inversion

**Figure 5:**
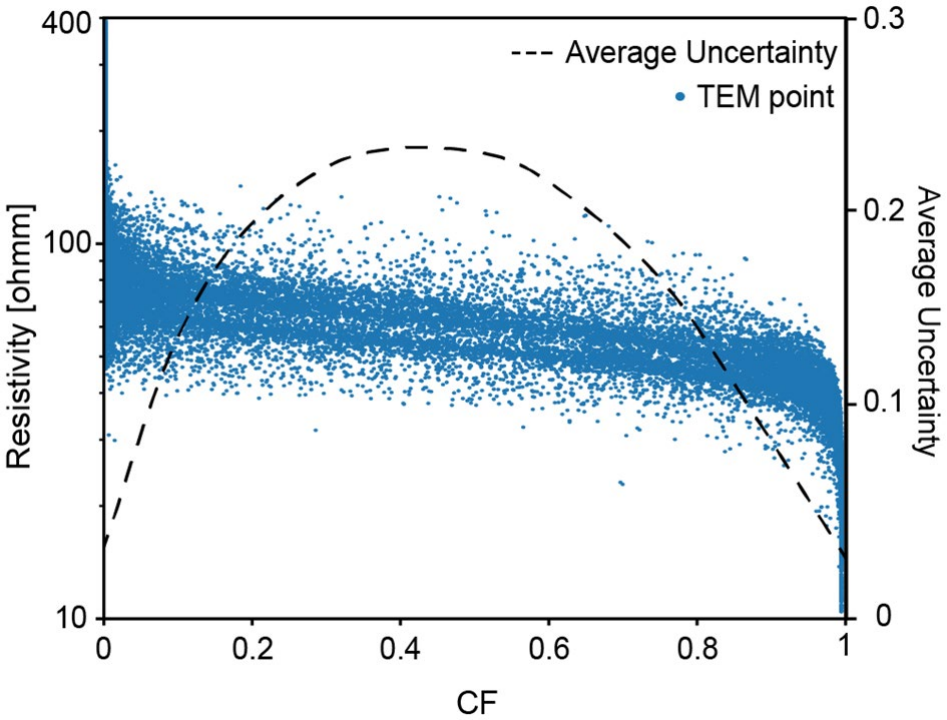
Results of the inverted translator function on 50’000 TEM points. In addition, the averaged uncertainty calculated with a moving mean of 0.05 CF width.

The average data-misfit [see Eq. (2)], or residual, of the application of the translator function to the geophysical models is 0.38. It means that the difference between the predicted $CF_{rho}$ and $CF_{log}$ is more than two times within the uncertainty range. The main source of misfit is the resolution issue. Because of the footprint of the geophysical equipment and of the damped least-squares inversion performed on the TEM data, we tend to have a smoother transition in the geophysical data than the reality. Since we are comparing the results with borehole data, which tend to reflect these sharp transitions, they can be a source of misfit between the predicted $CF_{rho}$ and the actual $CF_{log}$. Some thin layers of resistive materials in a large conductive layer will not be caught by the resolution of the equipment but will increase the residual value. However, the main advantage of the^14^ methodology is that the translator function adapts, to counterbalance these effects. Figure 5 shows the results of the inverted translator function and the averaged uncertainty on 50’000 TEM points. Most of the uncertainty is concentrated on the central points that present an intermediate resistivity since small uncertainty on the resistivity in this area will affect strongly the predicted CF. The range of values in which the transition between clay to non-clay highest probability happens is between 90 and 40 Ohms. Such values are coherent with the expected manual interpretation since Aare Quaternary Clay’s have a resistivity between 10 and 50 Ohms. The models are blinded at the standard depth of investigation obtained from the resistivity models and are then passed to the geostatistical simulation step.

### 3D clay model

**Figure 6:**
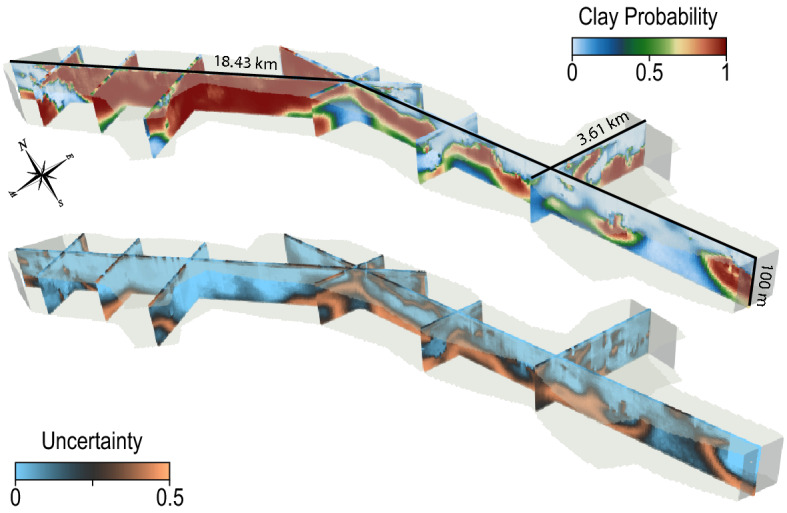
3D view of slices through the clay fraction model, with the associated uncertainty. The z-scale is exaggerated.

The results presented here are based on 200 simulation loops of 10 realizations each. For each loop, a new TI and HD set are generated with a GRF simulation, and a different random subset is drawn for the cross-validation. The groups were constituted using a K-Means algorithm of the spatial coordinates of the CF data. As mentioned before, the *Deesse* algorithm is controlled by three main parameters: the scanned fraction, the threshold, and the number of neighbors. The TI scan fraction is common to all variables and was set to 5%. The pattern will be compared to the TI until the scanned fraction is reached or until the threshold is respected on all variables. The threshold is set to 10% on three neighboring nodes for the auxiliary variable and 1% on 24 nodes for the main variables. If the threshold value is never reached, the best candidate is selected and the point is flagged to be re-simulated at the end of the simulation. The scanning path in the TI is random. The result is a set of 2000 simulations, based on 200 different TI. The resulting 3D model has a cell dimension of 50 m by 50 m, and a vertical resolution of 2 m. The surface covered is 35 km^2^, for a computational time of 10 h on a CPU cluster. Vertical cross sections of the averaged model are displayed in Fig. 6. A clear trend in the model is present between the northern and the southern side of the model. Such variation was expected due to the variations in the shape of the valley and is corroborated by the boreholes. The uncertainty of the data is higher in the deeper cells of the model, where no boreholes and no geophysical data are present to constrain the simulations. They also denote the transition zones between clay and non-clay areas and reflect the uncertainty on the exact depth of transition. The shape of the underground structures is consistent with existing Quaternary deposition conceptual processes. However, the visual consistency is not sufficient to trust a model.

**Figure 7:**
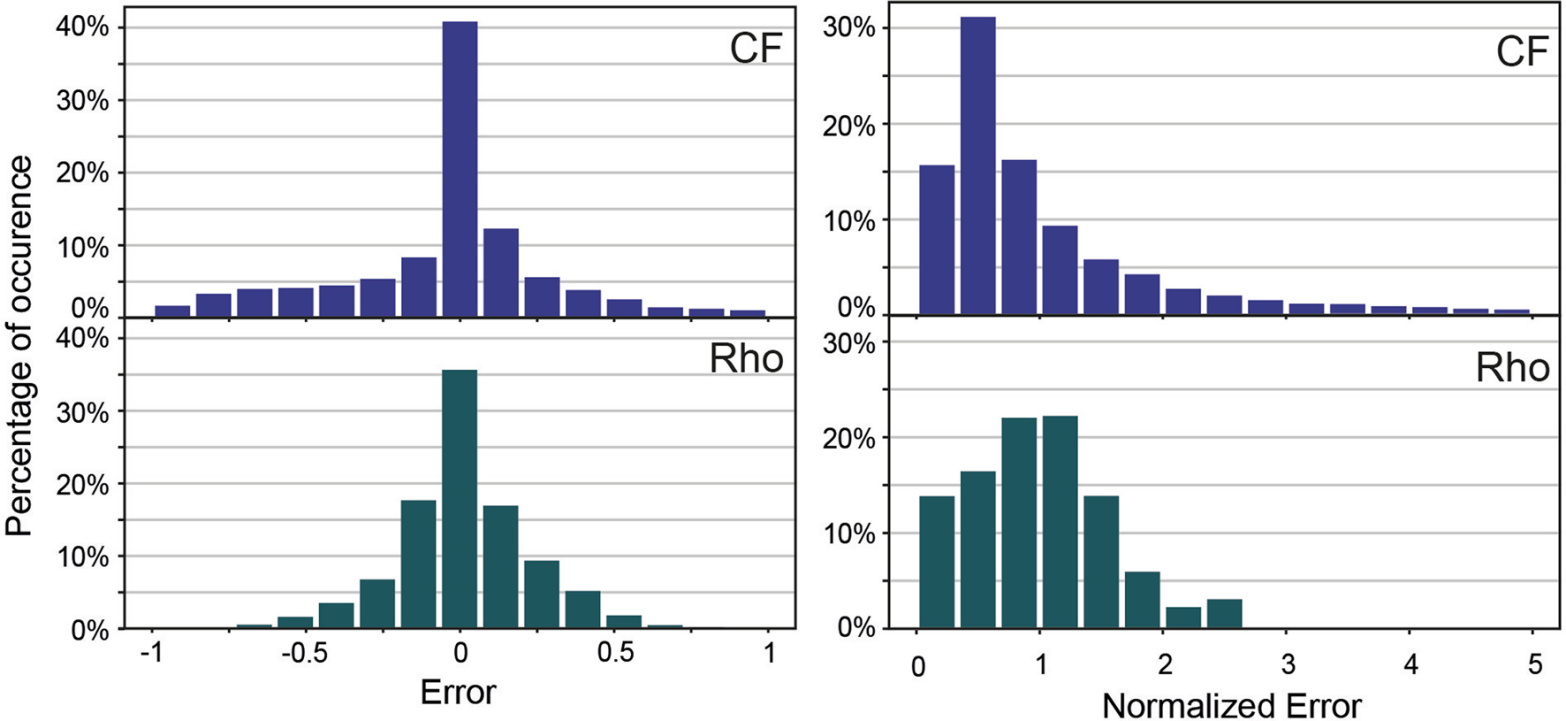
Histogram of the errors and the normalized error for the two main variables: Resistivity (Rho) and clay fraction (CF).

The error and the normalized error distributions [see Eqs. (6) and (7)] calculated with cross-validation are displayed on Fig. 7. During the simulation, the two main variables are the resistivity and the clay fraction. The error estimation during the cross-validation step is performed on both. The error is centered on zero for the two variables, suggesting that we tend to predict a correct value with no bias on average. Furthermore, in respectively 44% and 37% of the cases, we managed to predict a value that was in the 10% range of the real value. In terms of normalized error, the mean of the normalized error for resistivity is 0.98 ($\sigma = 0.56$). Such a distribution suggests that the standard deviations of the simulations are in the same order of magnitude as the real error. The uncertainty is therefore well predicted. On the other hand, the mean of the CF normalized error is 0.89 ($\sigma = 0.5$), meaning that we tend to slightly overestimate the error in the simulated data compared to the real one. But overall, the cross-validation indicates that on average the correct value is simulated with a standard deviation reflecting well the possible uncertainty. A strong result is that the resistivity is predicted accurately, showing that this method can be of great use even to only interpolate resistivity maps.

### Comparison with existing model

In an effort to homogenize and digitize all geological data from the Quaternary formations in Switzerland, the *GeoQuat* project made a prototype and demonstration study on the Aare Valley. In this context, a deterministic geological model of the area was done using a manual interpretation of boreholes, of geophysical data, and geological knowledge through the use of geological cross sections^39^. When comparing the two models, we should keep in mind that new data are included in the stochastic model that was not available at the time of the construction of the deterministic model: even if the boreholes database is the same, the tTEM data were only acquired in 2020. After incorporating the boreholes, they manually correlated units and facies and used the nearest neighbor interpolation to extend the model to a full 3D volume^39^.

**Figure 8:**
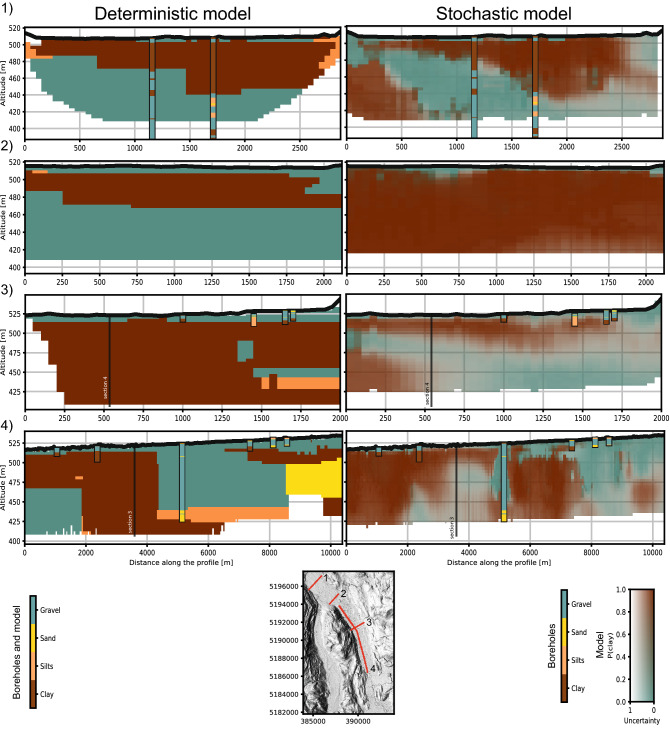
Comparison between existing geological model and this study model. The position of the sections 3 and 4 intersection is outlined.

Figure 8 shows the comparison between a few cross-sections in the deterministic model and in the model generated with MPS. The deterministic geological model and the boreholes are displayed using their USCS primary components. The transparency of the MPS model reflects the uncertainty. Higher is the transparency, higher is the associated uncertainty. Both models are in agreement with the borehole data at least for large-scale structures. We can see that the MPS-generated geological model displays structures that are more realistic geologically speaking. Some comments can be made for the cross-sections:

- Sections 1 are in agreement. The model is well constrained by two deep boreholes, going all the way through the model. Even if the nearest neighbor interpolation of the deterministic model displays some unrealistic blocky shapes, the global structures are similar between the two models.
- Sections 2 are drastically different between the deterministic and the stochastic model. The absence of boreholes to constrain the deterministic model causes the propagation of a gravel layer from distant boreholes, that is most probably not present at this location. The inverted resistivity calculated from the tTEM data is low and is most probably associated with thick clay bodies. Such a section shows improvement in the models, because of the additional data.
- Section 3 illustrate the importance of uncertainty. No tTEM data has been acquired in the area, and both models rely on the same data. The upper few meters of the models are well identified as being the upper gravel body on both models. However, the use of the proposed method revealed a possible resistive (sand or gravel) body deeper. The uncertainty is high, as highlighted by the color range. The presence of this layer cannot be established for sure. Although it is likely a possibility that has to be considered when assessing the area.
- Sections 4 show how a local Quaternary structure can drastically affect the deterministic model. The 100m deep borehole in the center of the cross-section indicates a gravel layer that is abnormally thick compared to the other nearby boreholes in the section. This over-thickening is extremely local and is most probably due to a gravity mass movement a few dozen thousand years ago according to personal communication with a Quaternary geologist. Because of this special borehole, the deterministic geological model overestimated the thickness of the upper aquifer over a very wide area.

**Figure 9:**
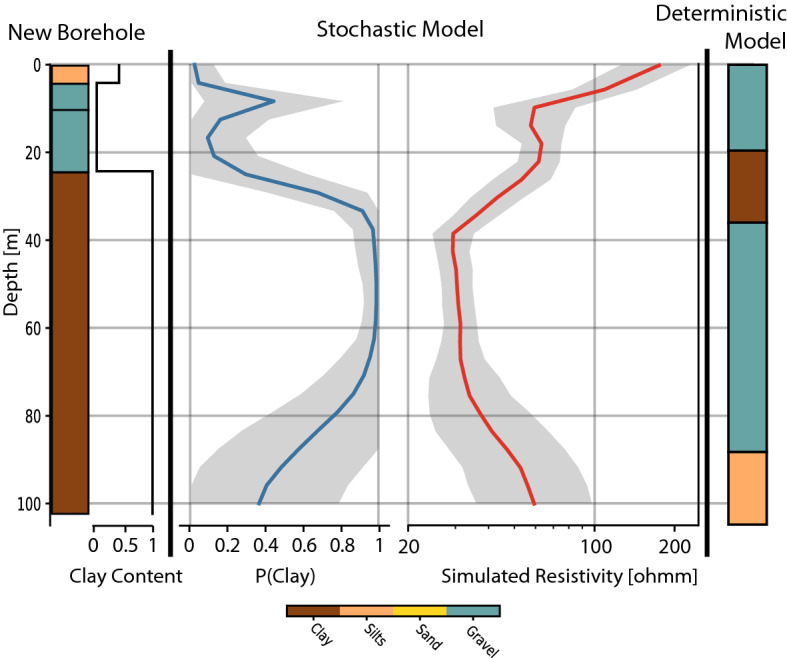
Comparison between a new borehole in the village of Wichtrach (not included in any of the two models), and the corresponding cell in the stochastic model and the deterministic geological model.

Finally, we could test the quality of the model by comparing the geology observed in several newly made boreholes that were not taken into account in any of the two models because they were drilled recently. The new shallow boreholes (max 10m) agree well with both models at 85% and 94% respectively for the deterministic and stochastic models. These results are not surprising since the upper gravel layer is more or less present in the whole domain. Most of the errors are due to some minor over- or underestimation of the depth of transition. However, a small number of deep boreholes were drilled too. In Fig. 9, a new borehole log is compared to the two models. The borehole is situated in the middle of the Wichtrach village, where no tTEM data can be acquired. The stochastic model reflects well the geology observed in the borehole. The stochastic model predicts the presence of the gravel aquifer on top and high resistivities like the deterministic model up to a depth of around 25m. But then the stochastic model predicts the presence of a massive clay layer up to a depth of 80 m before indicating a large uncertainty at a deeper depth. This prediction matches well the the geological observations well, which also show a massive clay layer. On the contrary, the deterministic model predicted a massive gravel layer just below a thin clay layer, followed by silts. This is not what has been observed in the borehole. Both models are constrained by the same boreholes data around, but while the stochastic model infers the regional trend by integrating all data of the area, the deterministic model is only constrained by nearby points and cross sections.

## Discussion

In this study, we presented a methodology that permits propagating the uncertainty all the way from the geophysical data and borehole logs to the final model of the clay fraction. In addition, all input data are used as hard data within their uncertainty range. A noisy data point in the field will lead to a more uncertain cell in the model. This feature is a key point to obtain a robust error estimation on the final model. Furthermore, being able to integrate uncertain boreholes due to poor or absent descriptions, unsure positioning, or missing data is a great advantage. The use of MPS makes it possible to generate models presenting complex patterns that are impossible to reproduce using approaches such as kriging. This method also has the advantage of relying on the data itself to deduce spatial patterns without any prior knowledge. Of course, this is only applicable to regions where the data coverage is sufficient. However, in a region with sparser data coverage or to increase the quality of the model, prior knowledge can also be added through the use of auxiliary variables. After the data preparation, the generation of high-resolution valley scale models can be achieved in a few days compared to months for deterministic ones.

In terms of geophysical technique, the application of tTEM in the upper Aare valley has proven that the technique is reliable and capable of providing relevant information regarding the 3D structure of the underground. This is confirming the previous conclusion from Sandersen et al.^46^. But, in addition, we argue that a proper and efficient way to integrate these types of large 3D datasets with borehole data can only be achieved through automatic methods. We also expect that the proposed methodology could be very useful for the integration of dense airborne data sets.

However, the estimation of lithology from resistivity has also its limitations. First of all, if the area presents significant salinity variations, it may introduce non-uniqueness. The same resistivity can correspond to saturated resistive material or clay. We would then only rely on boreholes to identify the nature of the underground. The proposed method would need to be extended in order to include a 3D estimation of the salinity as an additional auxiliary variable. This may be possible using additional hydrogeological information. Furthermore, the proposed method is efficient to distinguish between resistive layers (sand or gravel) and conductive layers (clay), but the resistivity difference between sand and gravel does not allow the method to distinguish them clearly. That is the main reason why we decided to focus on the identification of the clay layers only.

Even without simulating the CF value, our approach should also be considered when interpolating 1D or 2D resistivity models to 3D, especially when dealing with sharp inversion. Traditionally, this is done using Kriging. But kriging being the best unbiased linear estimator, it leads to a smooth interpolation between sharp models and inevitably adds artifacts in the 3D resistivity models. In the context of Quaternary deposits, the range of the variogram can sometimes be smaller or equivalent to the distance between two acquisition areas, leading to a wrong or incomplete interpolation. The proposed method has the advantage of relying on the 1D soundings directly instead of a grid made by kriging and it will interpolate between the models with a coherent sharpness. A similar methodology proposed by Vilhemlmsen et al.^15^ relies on an indicator kriging on the cluster value with a short range to fill the gaps between the acquisition lines in the TI, causing possible over smoothing. The methodology presented here has the advantage of not relying on any pre-interpolation of the data before the simulation.

Of course, the inversion itself is already an interpretation of the data, and in addition, the uncertainty associated with the geophysical inverted models is often difficult to estimate exactly. A further improvement could be the development of an algorithm that would work on the geophysical measurements directly, without any inversion, instead of using inverted models. By doing so, we could be able to compare directly the errors between the real field data and the final CF model. It would also simplify the workflow by avoiding a double inversion, once for the resistivity model itself and once for the CF algorithm.

The comparison between the existing model and the automatic model (Fig. 8) shows the risk of using a deterministic approach. On cross-section 2, the deterministic model predicts a thick gravel body at about 40 m depth. This body was placed there only relying on boreholes that are hundreds of meters away (close to Cross-section 1), and after including geophysical data we are now confident that there is no such body there. Of course, it was an interpretation based on the data available at that time, but no indication of uncertainty was available. In this context, taking decisions based on such kind of model is risky and should be avoided.

An increasing number of countries are developing centralized databases to host geological-related measurements. In this context, the use of an agile and reliable data aggregation algorithm is a promising approach. Being able to deduce spatial patterns from data without or with little prior knowledge prevents the infusion of structures from arbitrary choices, which is usually done during a manual interpretation of geophysical models. Standardization of description methods through different drilling contractors and open-access data are the key points to make this method application fast and more adaptable to new field areas.

An improvement of the method could be to forecast permeability values. If permeability measurements are conducted, either by using packing or pumping tests, a new variable could be added to the simulation to directly generate permeability fields. Another possibility would be the definition of a coupled probability density function between resistivity, clay fraction, and permeability from a set of prior knowledge or field knowledge. We would then be able to directly generate parametric fields. Finally, as mentioned earlier, we think that the interpolation methodology introduced here could be applied to other 3D geophysical models, in order to fill partial maps. Often, due to field constrain (inaccessible areas, corrupted data, different acquisitions,...) some areas are less densely covered than others. Using non-deterministic MPS, with Gaussian Pyramids, complete and homogenous parametric models could be generated with proper uncertainty quantification.

## Conclusion

In this paper, we showed that a novel workflow combining the clay fraction estimation algorithm^14^ with our modified Multiple Points Statistic algorithm is a robust method for the automatic generation of a 3D clay fraction model. The resulting 3D models can be used by local authorities or project managers to better plan the development and underground use of the region. These models can be used for example to locate potentially highly permeable zones for groundwater exploitation, or geothermal developments, or to evaluate the potential presence of construction materials in the underground. Because the method provides uncertainty estimates it can also help design the acquisition of further data.

Our method has the advantage of being data-driven and not relying on manual interpretation of the structures. The workflow is automatic and includes: (1) the inversion of the translator function on the resistivity models and the borehole data, (2) the automated fit of the variogram used for the GRF model, and (3) the generation of different TI and HD for each MPS simulation. The user has only to select a few parameters such as the number *n* of neighbors, or the threshold *t* for the MPS simulations, but default values can be used. Furthermore, it reproduces structures that cannot be modeled using classical two-point interpolation such as kriging. By comparing the geology observed along newly made boreholes with our model, this study always predicts well the general trend as shown for example in Fig. 9. The workflow can easily be incorporated with public databases, allowing the authorities to update automatically the 3D model regularly when new field data become available.

Finally, the paper also proves the efficiency of the towed Transient ElectroMagnetic (TEM) method. When most of the previous publications used airborne TEM data, we integrated towed TEM data. This allowed us to reach a resolution in space and depth that is unachievable with any other geophysical method at the moment. Such data acquisition should be increasingly considered, even for medium or small-scale problems. Easy, fast, and cheap integration of multiple, often existing, data types can only be beneficial whatever the purpose.

## Data Availability

The tTEM data used in this paper are available from the open access archive Zenodo at the following URL: 10.5281/ZENODO.4269887. The data set is documented in Neven et al.^26^. The borehole data set is available from the Swiss Federal Office of Topography (Swisstopo) but restrictions apply to the availability of these data, which were used under license for the current study, and so are not publicly available. Data are however available from Swistopo upon reasonable request.

## Code availability

The geostatistical codes used in this study are implemented in the python *Geone* package freely available at https://github.com/randlab/geone. To use all the functions of DeeSse, an academic license can be freely obtained. The CF model will be freely available.
